# Large Infected Abdominal Wall Abscess Following a Minor Mechanical Strain: A Case Report

**DOI:** 10.7759/cureus.91846

**Published:** 2025-09-08

**Authors:** Razan Honeini, Rawan Honeini, Alaa Albadwy, Mahmoud Abughazal, Noor Ali

**Affiliations:** 1 Emergency Medicine, United Lincolnshire Hospitals NHS Trust, Grantham, GBR; 2 Emergency Medicine, United Lincolnshire Hospitals NHS Trust, Boston, GBR; 3 Emergency Department, United Lincolnshire Hospitals NHS Trust, Grantham, GBR

**Keywords:** abdominal lump, incision and drainage (i&d), intraabdominal abscess, neutrophilic infiltration, periumbilical mass

## Abstract

We present a case of an 85-year-old female who developed an acute abdominal lump and pain after pulling herself up from a car seat. She experienced a sudden "pop" sensation, followed by a palpable periumbilical lump. Imaging revealed a large, infected collection in the left anterior abdominal wall. The patient underwent urgent incision and drainage, which was completed successfully without complications. Histological analysis of wound discharge confirmed acute inflammation without malignancy. This case underscores the need for early recognition and intervention in elderly patients presenting with an acute abdominal lump and pain.

## Introduction

Abdominal wall abscesses are often associated with previous surgeries, trauma, or underlying infections. However, the sudden development of a large infected collection following minor mechanical strain is uncommon, particularly in an elderly patient with no prior history of hernias. Most abdominal wall abscesses arise due to direct inoculation (e.g., surgical site infections), hematogenous spread from distant infections, or extension of intra-abdominal sepsis such as appendicitis or diverticulitis [1]. Previous abdominal surgeries and known hernias are major predisposing factors due to structural weakness and impaired tissue perfusion [2]. Patients with comorbidities such as diabetes mellitus, peripheral vascular disease, or immunosuppression are at increased risk of more severe infections and complications [3]. This case highlights the importance of early imaging and surgical intervention to prevent complications, especially in vulnerable populations.

## Case presentation

An 85-year-old female, known to have hypertension, hypothyroidism, and osteoarthritis, with no previous history of abdominal hernias, presented to the emergency department with complaints of abdominal pain and a periumbilical lump. She reported experiencing a sudden "pop" in the left upper quadrant of her abdomen after pulling herself up from a car seat three days prior to admission. The pain was of low grade (scoring 2 out of 10 on the Numeric Rating Scale at rest). She noted that applying direct pressure alleviated the pain, while movement aggravated it. There was no radiation of pain, and her symptoms remained constant over the three days.

On examination, she was alert, with a National Early Warning Score (NEWS) of 0. Her vital signs were stable: heart rate of 82 bpm, respiratory rate of 16 breaths per minute, temperature of 36.6°C, blood pressure of 167/68 mmHg, and oxygen saturation of 97% on room air. A firm, erythematous, warm-to-touch, and irreducible lump measuring approximately 10 × 8 cm was palpated in the left upper quadrant.

Blood tests (Table 1) revealed a significantly elevated C-reactive protein (CRP) of 236 mg/L and a white blood cell count (WCC) of 16.6 × 10⁹/L, suggesting an ongoing inflammatory or infectious process.

**Table 1:** Lab parameters. Hb: hemoglobin; WBC: white blood cell count; Cr: creatinine; Na: sodium; K: potassium; CRP: C-reactive protein; GFR: glomerular filtration rate; ALT: alanine transaminase.

Lab parameters	Value at presentation	Value 2 days post operation	Normal range
WBC	16.6	12.7	4.5-11 x109/L
Platelets	316	345	150-400 x103/µL
Urea	4.2	3.9	2.1 to 8.5 mmol/L
Cr	61	58	59-104 μmol/L
Na	136	140	136 to 145 mmol/L
Hb	11.8	11.7	14 to 18 g/L
K	4.2	3.6	3.6-5.2 mmol/L
CRP	236	47	Below 5 mg/L
GFR	81	82	90-100 ml/min
Bilirubin	25	20	0-21 μmol/L
ALT	45	43	0-33 U/L
Alkaline phosphatase	108	107	30-130 U/L
Neutrophils	13.85	9.46	2.1-7.4 x109/L

**Figure 1:**
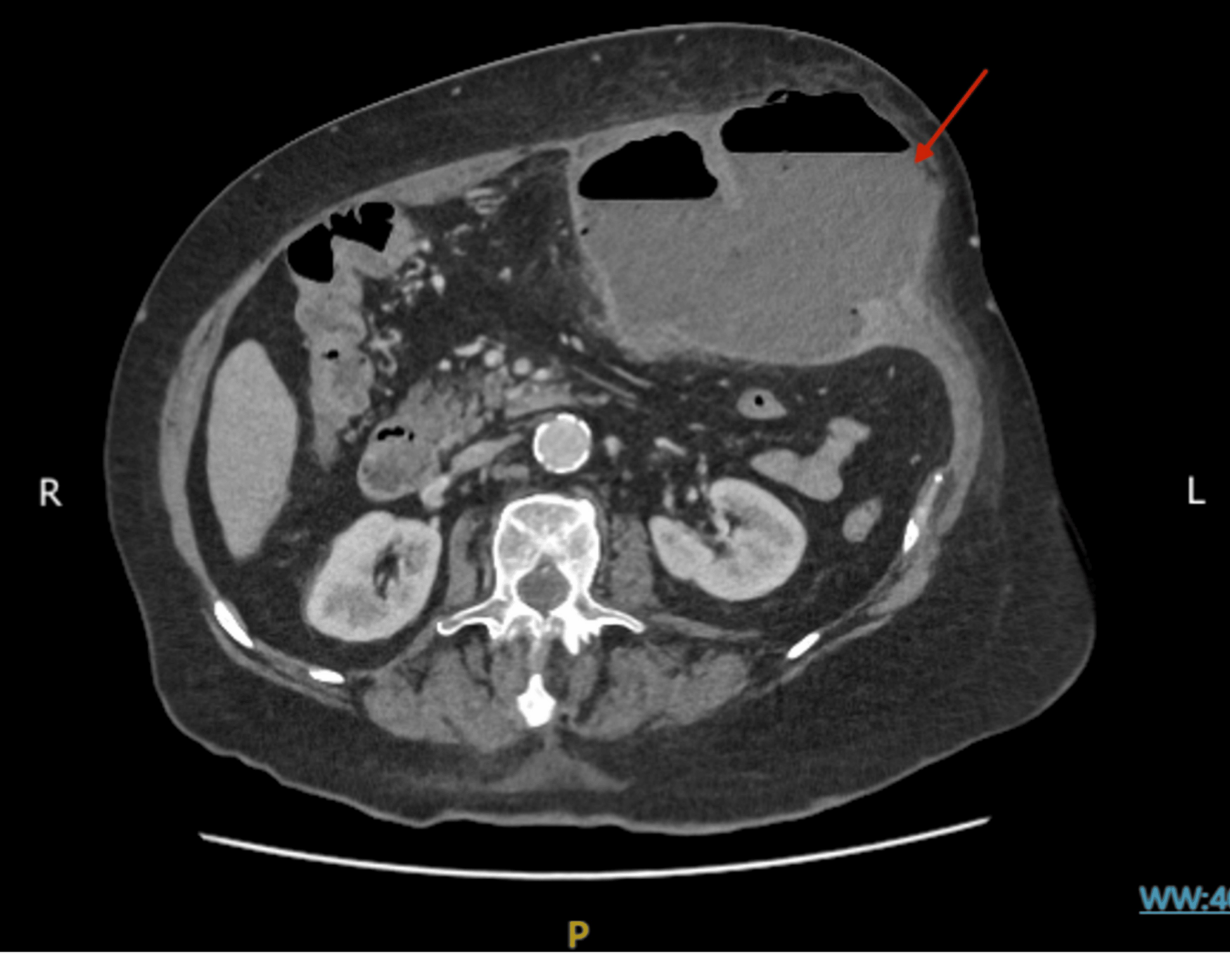
Axial contrast-enhanced CT scan showing an infected collection in the left anterior abdominal wall (red arrow).

**Figure 2:**
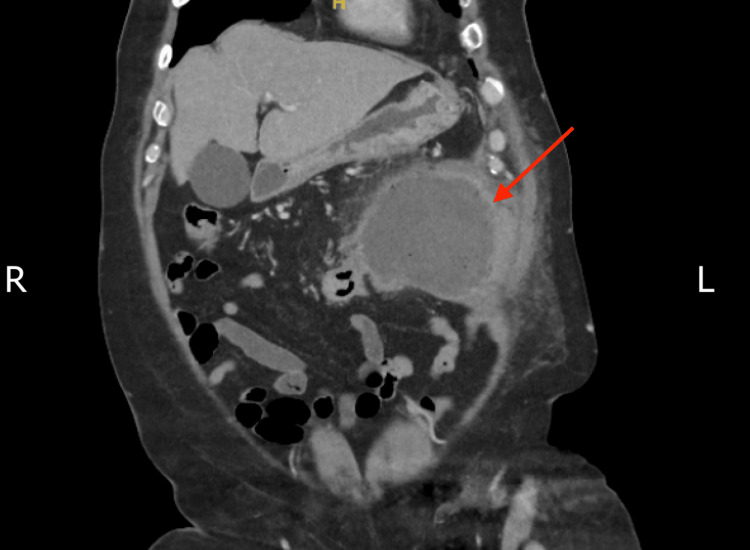
Coronal contrast-enhanced CT scan showing an infected collection in the left anterior abdominal wall (red arrow).

A contrast-enhanced CT scan of the abdomen showed a large, well-defined, lobulated collection with air-fluid levels in the left anterior abdominal wall, measuring 14.5 cm craniocaudal × 14 cm transverse × 11 cm anteroposterior. The collection was in close proximity to the adjacent transverse colon, with a possible communication between the abscess and the bowel. Mild nonspecific thickening of the transverse colon was noted (Figures 1, 2).

Given the significant findings on imaging, the patient was referred to the surgical team, who performed an urgent incision and drainage (I&D) of the abdominal abscess after two days. The procedure was successfully completed without complications. Wound discharge samples were sent for histological evaluation, and cytological preparation and H&E staining of the clot revealed numerous neutrophils and occasional macrophages, consistent with acute inflammation. No atypical or malignant cells were identified. Microbiological culture revealed moderate growth of *Streptococcus constellatus*.

During her postoperative stay in the surgical ward, her blood tests were regularly monitored, and she was treated with intravenous antibiotics (ciprofloxacin and metronidazole). She showed progressive clinical improvement, with downtrending inflammatory markers (Table 1) and no signs of recurrent infection.

## Discussion

The development of an infected abdominal wall collection following minor mechanical strain is a rare clinical occurrence. While soft tissue infections are common, the emergence of a significant collection, particularly one potentially communicating with the transverse colon, necessitates consideration of underlying gastrointestinal or structural pathologies.

Spontaneous perforation of the colon, although uncommon, may present insidiously and be mistaken for soft tissue infections, especially when extraluminal gas or feculent material tracks through fascial planes. Undiagnosed diverticular disease is a well-documented cause of spontaneous colonic perforation, often presenting as a localized abscess or phlegmon in atypical sites, including the anterior abdominal wall [4,5]. Ischemic colitis is another differential, where compromised blood flow can lead to mucosal necrosis, ulceration, and ultimately perforation, particularly in watershed areas of the colon [6].

Infection of a previously unnoticed hernia sac or occult abdominal wall defect can become evident following increased intra-abdominal pressure or mechanical strain. Such strain may precipitate incarceration, strangulation, or rupture of a hernia sac, with subsequent infection and abscess formation. These complications, though infrequent, have been documented especially in elderly or chronically ill patients [7,8].

Subclinical abdominal wall cysts, hematomas, or seromas may rupture following trauma or sudden mechanical stress. These sterile collections can become secondarily infected, mimicking intra-abdominal pathology. The transformation of a chronic hematoma into an abscess is a recognized but underreported complication [9,10].

The large size of the collection and radiological suggestion of colonic communication in this case warranted early surgical exploration. Incision and drainage remain the cornerstone for managing such infections, and histopathological analysis plays a critical role in excluding malignancy or chronic granulomatous disease [1]. In this case, histology confirmed acute inflammation with no neoplastic changes, highlighting the importance of tissue sampling in atypical presentations.

In this case, the integration of clinical data, laboratory trends, and imaging findings was central to diagnostic decision-making. The persistently elevated inflammatory markers (WBC and CRP) alongside the large, irreducible abdominal wall collection raised suspicion of a more complex underlying pathology than a simple soft tissue infection. The CT features further guided the differential toward an infection tracking from an intra-abdominal source. The subsequent postoperative downtrend in inflammatory markers, in parallel with clinical recovery, supported the accuracy of surgical intervention and excluded alternative differentials such as malignancy or chronic granulomatous disease.

## Conclusions

This case highlights the importance of early imaging and surgical evaluation in elderly patients presenting with acute abdominal swelling and pain, even when preceded by seemingly minor trauma. CT imaging played a key role in defining the extent of the collection, identifying potential intra-abdominal communication, and guiding timely surgical decision-making. Prompt incision and drainage, supported by histological and microbiological analysis, were essential not only for effective treatment but also for excluding serious underlying conditions such as malignancy or chronic inflammatory disease.

In elderly or comorbid patients, minor mechanical events can unmask occult pathologies, including infected hematomas, ruptured cysts, or complications of undiagnosed gastrointestinal disease. Early recognition, multidisciplinary input, and timely intervention remain critical to reducing the risk of systemic sepsis, bowel perforation, and prolonged recovery. This case reinforces that surgical drainage, combined with thorough pathological evaluation, remains the cornerstone of safe and effective management in such atypical presentations.
